# Effects of planting density and row spacing configuration on dry matter accumulation and distribution, nutrient content, and yield in cotton

**DOI:** 10.3389/fpls.2026.1797286

**Published:** 2026-05-05

**Authors:** Ziwei Bai, Nan Cao, Ming Wen, Qiang Hu, Yulong Wang, Jiao Lin, Sumei Wan

**Affiliations:** 1 College of Agriculture, Tarim University, Alar, China; 2 Key Laboratory of Genetic Improvement and Efficient Production for Specialty Crops in Arid Southern Xinjiang of Xinjiang Corps, Tarim University, Alar, China

**Keywords:** dry matter accumulation and distribution, nutrient content and distribution, planting density, row spacing, yield formation

## Abstract

**Background:**

Dry matter accumulation and distribution constitute the essential material basis for crop yield formation, directly impacting both yield and quality development. Nonetheless, the effects of planting density on dry matter accumulation, distribution, and nutrient uptake in cotton across various row spacing configurations remain ambiguous.

**Methods:**

This study conducted a two-year field experiment utilizing the cotton cultivar “Tahe 2”. In 2024, a single-factor trial was adopted with three row spacing treatments: same row spacing (S3), wide-ultra-narrow row spacing (S4), and wide-narrow row planting (S6). In 2025, a two-factor split-plot design was used: main plots were the three row spacing patterns (S3, S4, S6), and subplots were three planting densities: 135 000 plants ha^-1^ (D1), 180 000 plants ha^-1^ (D2), 225 000 plants ha^-^¹ (D3). We investigated leaf area index (LAI), dry matter accumulation and distribution, nutrient content, and yield.

**Results:**

In 2024, S6 showed the highest leaf area index (LAI) and dry matter accumulation (*p*< 0.05). In 2025, LAI and dry matter accumulation increased significantly with increasing plant density (*p*< 0.05). The dry matter partitioning ratio to reproductive organs increased with growth. Row spacing and planting density significantly affected leaf nutrient contents; upper-leaf nitrogen (N) and potassium (K) first increased then decreased, while middle- and lower-leaf N and leaf phosphorus (P) at all positions declined gradually. Boll number per plant decreased with increasing density, whereas yield was significantly affected by their interaction (*p*< 0.05). The highest yield was found in S6 in 2024 and S6D2 in 2025. Two-year experiments indicated that S6 combined with 180,000 plants ha^-^¹ optimized population structure, improved resource use efficiency, and increased cotton yield.

**Conclusion:**

Among the three row spacing configurations tested, the medium planting density of 180,000 plants ha^-1^ under the wide-narrow row planting pattern (S6) optimized the leaf area index, enhanced population dry matter accumulation, and improved distribution to reproductive organs, thereby maximizing seed cotton yield, which reached up to 6420.47 kg·ha^-1^.

## 1 Introduction

Cotton (*Gossypium hirsutum* L.) is a vital economic crop and an essential raw material for fiber, holding a prominent position in China’s national economy and textile industry (Feng et al., 2022). Xinjiang has emerged as the primary region for cotton production in China. Recent studies indicate that Xinjiang contributes 80% to 90% or more of the nation’s total cotton output, establishing it as a critical national supply base for this commodity (Kang et al., 2023; Zhou et al., 2025, 2024). Southern Xinjiang, recognized as a key area for high-quality cotton production, significantly contributes to regional economic development (Zhang et al., 2024). Most studies on cotton planting density and row spacing in Xinjiang have focused on the one−film six−row system (Liu et al., 2024; Yang, 2024). To improve defoliation efficiency, yield, and quality, one−film three−row and one−film four−row patterns are used in production (Liu et al., 2024; Wang et al., 2023, 2024). However, the optimal planting density under these systems remains unclear, and research on cotton growth, development, and nutrient status is limited and worthy of further investigation. This study is crucial for enriching cotton cultivation techniques in southern Xinjiang and promoting high−quality and efficient cotton production.

Cotton production in Xinjiang typically employs a high-density, mechanized-harvest cultivation strategy. The dense canopy inhibits the penetration of defoliant droplets to the lower-layer leaves, leading to uneven defoliation and an increased risk of impurity contamination during mechanical harvesting (Wang et al., 2019; Xin et al., 2018). Consequently, recent studies have introduced the equal-row-spacing planting pattern. Compared to the wide-narrow row configuration, an optimal equal-row-spacing layout can enhance the canopy structure and improve light interception within the cotton population. This adjustment increases the efficiency of solar radiation utilization and subsequently boosts the boll number per plant, boll number per unit area, and overall yield performance (Gao et al., 2024; Hu et al., 2021; Zuo et al., 2023). Excessively wide row spacing can increase the number of bolls per plant; however, it often results in insufficient population density and decreased efficiency in the utilization of light and heat resources, ultimately failing to establish an optimal population structure (Gao et al., 2024; Hu et al., 2021; Zuo et al., 2023). More critically, a uniform row-spacing strategy struggles to sustain the dynamic balance between individual plant growth and overall population development throughout the growth period. An overly dense canopy can reduce the defoliation rate and increase the impurity content of machine-harvested seed cotton, thereby limiting the potential for synergistic improvements in yield and fiber quality (Gu et al., 2024; Lin et al., 2023). Consequently, optimizing plant and row spacing configurations not only facilitates the integration of agricultural machinery and agronomic practices, reduces labor input during harvesting, and enhances production efficiency, but also contributes to improved canopy structure and resource utilization, thereby enhancing both yield and quality in machine-harvested cotton (Hu et al., 2021).

The configuration of plant and row spacing in cotton production significantly influences essential agronomic traits, including plant growth, development, yield formation, and early-maturity performance (Zuo et al., 2023). Investigating various plant and row spacing configurations for machine-harvested cotton holds substantial importance for both cotton production and field practices (Gao et al., 2024). High crop yield and quality depend on substantial biomass production, and the high-yield potential of cotton is closely linked to population biomass production and its accumulation process. This accumulation relies on nutrient uptake, with a strong coupling observed between biomass and the accumulation, distribution, and translocation of nutrients such as nitrogen (Song et al., 2020). Numerous studies have shown that growth models such as the Logistic model can effectively describe the S-shaped dynamic processes of dry matter accumulation and nutrient uptake in cotton. These models provide a critical analytical tool for revealing the intrinsic relationship between the formation of cotton yield and quality, and the characteristics of biomass accumulation, distribution, translocation, as well as nutrient absorption and distribution. Therefore, investigating the effects of plant and row spacing configurations on biomass accumulation, distribution, translocation and nutrient uptake, partitioning and translocation in machine-harvested cotton is of considerable scientific and practical significance (Zhang et al., 2024).

Dry matter accumulation and allocation form the material basis for crop yield formation, directly affecting both yield and quality development (Liu et al., 2024). Research indicates that aligning suitable planting patterns with varietal architectural traits can enhance the processes of dry matter production and allocation. By optimizing plant density and row spacing configurations, one can improve canopy structure and light distribution, thereby increasing the photosynthetic productivity of the population and the translocation efficiency of assimilates to reproductive organs (Hu et al., 2021; Lakshmanan et al., 2025). Planting density plays a pivotal role in regulating dry matter accumulation and allocation. Generally, dry matter accumulation per plant declines as density increases. This leads to poor light distribution, reduced light transmittance to the lower canopy, and an imbalance in source-sink relationships. Such conditions can precipitate premature leaf senescence and diminish the sustained supply of assimilates, thereby constraining overall dry matter accumulation and its effective allocation to cotton bolls (Hu et al., 2021; Li et al., 2023). Moreover, excessive planting density is frequently linked to a reduction in boll weight or restricted boll development, ultimately undermining final yield formation (Khan et al., 2019).

Under identical planting density, the configuration of row spacing, such as wide-narrow row arrangements or optimized spacing structures, positively influences dry matter accumulation. Furthermore, fiber quality is either relatively less affected or shows no significant variation in response to changes in planting density and row spacing (Li et al., 2023; Zhang et al., 2024). Additionally, under the mechanized cotton harvesting conditions in Xinjiang, the integrated regulation of row spacing structure and planting density can synergistically enhance both yield and quality by improving photosynthetic production and optimizing indicators related to fiber quality (Zuo et al., 2024).

The accumulation of population biomass fundamentally relies on nutrient uptake and is closely linked to the nutrient absorption process (Iqbal et al., 2022). As a critical “source,” the functional maintenance and senescence of leaves in cotton directly affect carbon assimilation efficiency and the translocation of assimilates to cotton bolls. Maintaining an appropriate source-sink relationship and enhancing the population’s photosynthetic capacity are essential physiological foundations for promoting dry matter accumulation and ultimately increasing yield. Furthermore, rational planting patterns significantly impact the efficiency of resource acquisition and utilization within the population, thereby influencing both dry matter production and the allocation proportion to reproductive organs (Hu et al., 2021; Lakshmanan et al., 2025).

Rational planting density and row spacing configuration are critical agronomic practices for enhancing cotton population quality and achieving a synergistic improvement in both yield and quality. Numerous studies have shown that planting density and row spacing affect dry matter accumulation and its distribution among various organs by modifying canopy structure, light distribution, and source-sink relationships (Zuo et al., 2024). Both excessively high and low planting densities can result in yield losses. In contrast, an optimal planting density, when paired with well-designed row spacing, not only stabilizes and increases yields but also enhances resource use efficiency at the population level. Furthermore, this approach lays a technical foundation for reducing production costs and facilitating mechanized harvesting and large-scale production (Tang et al., 2025).

Selecting optimal row spacing and planting density is critical for balancing cost reduction and yield improvement. Although their individual effects have been widely studied, the interactive impacts on nutrient accumulation and cotton yield in Xinjiang remain poorly quantified, limiting the development of precision management strategies. We hypothesize that rational row spacing and planting density can enhance dry matter accumulation, nutrient uptake, and assimilate translocation to cotton bolls, thus increasing yield. A two-year field experiment was conducted to investigate dry matter partitioning, nutrient absorption, and yield formation.This study provides a scientific basis for precise cotton cultivation in arid and semi-arid regions, supporting the high-quality development of the cotton industry.

## 2 Materials and methods

### 2.1 Experimental site and design

**Figure 1:**
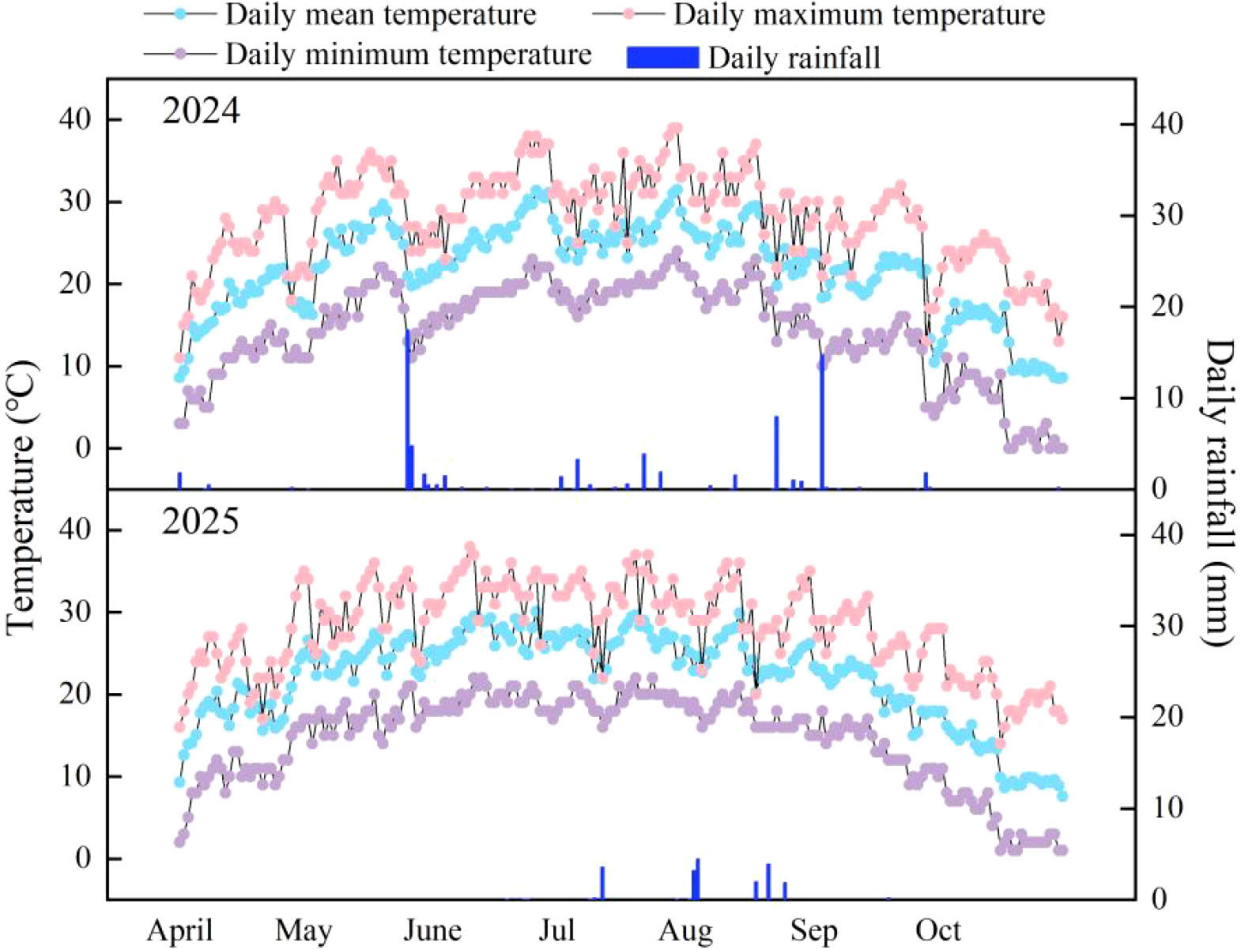
Meteorological data of the experimental area over two years.

This study was conducted from 2024 to 2025 at the Southern Xinjiang Industry-Education Integration Modern Agricultural Training Base in Alar City, Xinjiang. Alar City is characterized by a warm temperate, extremely continental arid desert climate, with annual solar radiation in the reclaimed area averaging between 133.7 and 146.3 kcal·cm^-^². The annual average sunshine duration varies from 2,556.3 to 2,991.8 hours, resulting in a sunshine ratio of 58.69%. The reclaimed area receives limited rainfall, negligible winter snowfall, and experiences significant surface evaporation. Annual precipitation averages between 40.1 and 82.5 mm, while annual evaporation ranges from 1,876.6 to 2,558.9 mm. The preceding crop in the experimental field was cotton. The soil texture was sandy loam with a bulk density of 1.56 g·cm^-^³, pH 7.50, total nitrogen content of 1.45 g·kg, available phosphorus content of 55.6 mg·kg, and available potassium content of 102.8 mg·kg. The air temperature and rainfall conditions of the experimental field are shown in **Figure 1**.

**Figure 2:**
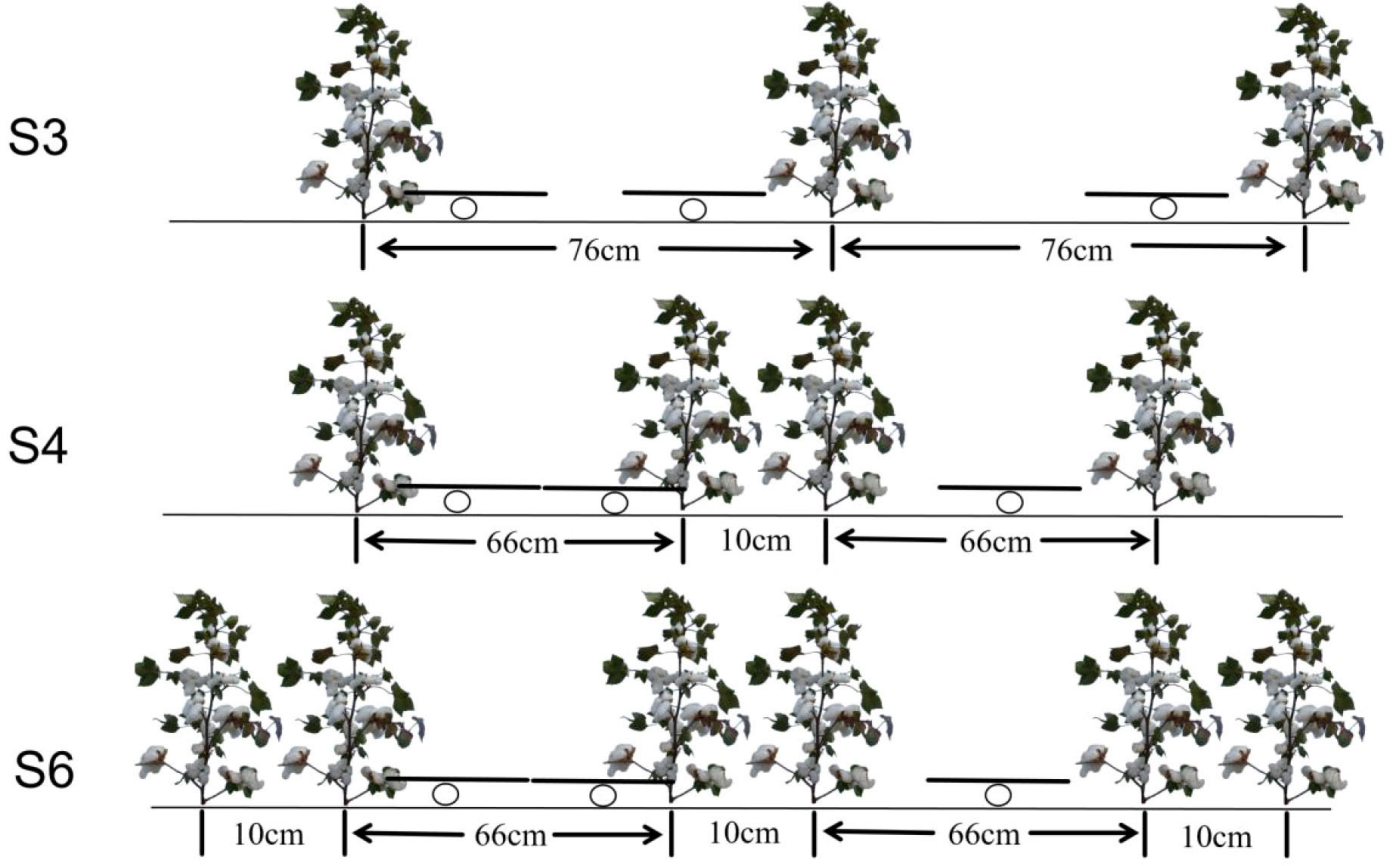
Schematic diagram of the cotton field planting pattern. The circles represent the drip irrigation tapes.

In 2024, a single-factor experimental design was utilized, incorporating three treatments: same row spacing (S3), wide and ultra-narrow row (S4), and wide-narrow row planting (S6), as depicted in **Figure 2**. Each treatment was replicated three times, resulting in a total of nine plots, each measuring 228 m² (20 m × 11.4 m).In 2025, a two-factor split-plot experimental design was employed, with plot configurations (S3: same row spacing, S4: wide and ultra-narrow row, S6: wide-narrow row planting) as the main plots and planting densities (D1: 135,000 plants ha^-1^, D2: 180,000 plants ha^-1^, D3: 225,000 plants ha^-1^) as subplots, with three replicates. The trial comprised 27 plots, each measuring 92 m² (4.6 m × 20 m). The three row spacings and planting densities were determined based on a review of the literature and field investigations of production practices.

In this study, a single-factor experiment (row spacing) was conducted in 2024, and a two-factor experiment (row spacing × planting density) was arranged in 2025, representing a progressive and goal-oriented scientific design. In 2024, the single-factor experiment was used to identify the main effect of row spacing and screen the suitable range of row spacing, laying a parameter foundation for subsequent experiments. In 2025, based on the above results, the planting density factor was introduced to systematically analyze the interaction mechanism between row spacing and density, and to optimize the configuration scheme of crop population structure. The overall experimental design follows a logic from simplicity to complexity, is rigorous and consistent with the research paradigm in the field of crop cultivation, which can guarantee the scientificity and practicality of the experimental results.

In the two-year experiment, the cotton variety “Tahe 2”, which is predominantly cultivated in southern Xinjiang, was selected. This cultivar has a growth period of 136 days, with stable and uniform growth throughout the entire growth stage. It features a cylindrical plant type and type-II fruit branches, with sturdy stems and an elegant plant architecture. The leaves are medium-sized with a dark green color. The cultivar exhibits a high percentage of pre-frost lint, smooth boll opening, and easy harvesting. It is an early-to-medium maturity, non-transgenic conventional upland cotton cultivar with resistance to Fusarium wilt and tolerance to Verticillium wilt, producing medium-staple fiber. It is suitable for cultivation in southern Xinjiang.

Seeds were manually sown at a density of two seeds per hole and at a depth of 2–3 cm. Following emergence, only one vigorous plant per hole was retained. All other management practices adhered to those typically employed in field production. Sowing occurred on 21 April 2024 and 20 April 2025, respectively, with manual thinning to the required density conducted on 18 May 2024 and 8 May 2025, respectively. The harvest dates were 20 October 2024 and 18 October 2025, respectively. Irrigation during the cotton growing season, along with the management of pests, diseases, and weeds, was performed in accordance with standard high-yield field practices.

### 2.2 Leaf area index

During the Seedling stage (S), Peak squaring stage (PSS), Peak flowering stage (PFS), Flowering and boll stage (FBS), Peak boll-setting stage (PBS), and Boll opening stage (BOS), three representative cotton plants were randomly selected from each plot. All leaves from each plant were clipped at the base, arranged on a black backdrop, and photographed to obtain images in JPG format. These images were then analyzed using image analysis software to derive leaf area measurements, from which the leaf area index was subsequently calculated. LAI was calculated as follows:


LAI = (Leaf area per plant × Number of plants per plot)/Plot area

### 2.3 Dry matter

At each growth stage of the cotton plants, three representative specimens were randomly selected from each plot. These specimens were categorized into lower (1st - 3rd fruit branches), middle (4th - 6th fruit branches), and upper (7th fruit branch and above) sections. The plant material was then divided into vegetative organs (roots, stems, leaves) and reproductive organs (buds, flowers, bolls). The samples were placed in envelopes and subjected to blanching at 105 °C for 30 minutes, followed by drying at 80 °C until reaching a constant weight. Finally, the samples were weighed, and the corresponding values were recorded.

### 2.4 Plant nutrients

At each growth stage of cotton, three representative plants were randomly selected from each plot. All leaves were detached from the base and categorized into lower (1st - 3rd fruit branches), middle (4th - 6th fruit branches), and upper (7th fruit branch and above) sections. The leaves were placed in paper envelopes, fixed at 105 °C for 30 min, and subsequently dried at 80 °C until reaching a constant weight. After grinding and sieving, the dry leaf samples were analyzed for nitrogen (N), phosphorus (P), and potassium (K) contents. Total nitrogen was determined using the Dumas combustion method with a Dumas nitrogen analyzer, total phosphorus was measured by the molybdenum-antimony anti-spectrophotometric method, and total potassium was assessed through flame photometry.

### 2.5 Cotton yield

Yield assessment occurs when the cotton boll opening rate surpasses 80%. During this evaluation, ten uniformly developed cotton plants are selected from each plot. These plants are then brought indoors for classification into three sections: lower (1st - 3rd fruit branches), middle (4th - 6th fruit branches), and upper (7th fruit branch and above). Cotton bolls are harvested from the designated sections, air-dried naturally, and weighed to determine the number of bolls per plant and the weight of each boll. Lint yield is computed by harvesting and weighing the cotton from each plot, which is subsequently converted to yield per hectare.

## 3 Results

### 3.1 Leaf area index

**Figure 3:**
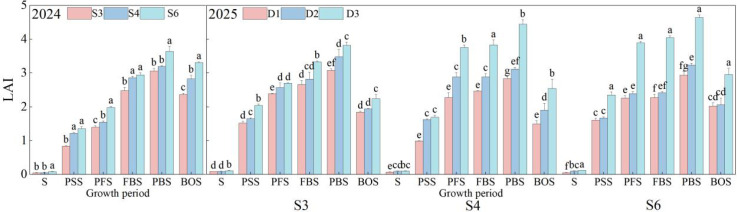
Dynamics of cotton leaf area index under different row spacing configurations and planting densities. S: Seedling stage; PSS, Peak squaring stage; PFS, Peak flowering stage; FBS, Flowering and boll stage; PBS, Peak boll-setting stage; BOS, Boll opening stage. Different letters indicate a significant difference at *p*< 0.05 among treatments.

LAI serves as a crucial indicator of canopy size, light use efficiency, canopy structural dynamics, and population quality in cotton. As illustrated in **Figure 3**, the LAI of cotton demonstrated a distinct pattern characterized by an initial increase followed by a decline throughout the growth stages. During the seedling stage, the differences in LAI among the three row spacing treatments were not statistically significant. From the seedling stage to the peak boll-setting stage, LAI increased rapidly, attaining its maximum at the peak boll-setting stage. During the boll-opening stage, LAI declined due to leaf senescence and shedding. In 2024, LAI consistently ranked in the order of S6 > S4 > S3 across all growth stages. The peak LAI values for all three treatments were observed at the peak boll-setting stage, measuring 3.64, 3.20, and 3.06 for S6, S4, and S3, respectively. The LAI of S6 surpassed that of S3 and S4 by 18.92% and 13.90%, respectively (*p*< 0.05). In 2025, LAI across the three row spacing treatments increased with rising planting density. The D3 density consistently exhibited the highest LAI throughout the entire growth period. At all three row spacings, LAI for each density increased rapidly prior to the peak boll-setting stage, reaching its maximum during that stage. At the peak boll-setting stage, LAI values across treatments were ranked as follows: S6D3 > S4D3 > S3D3 > S3D2 > S6D2 > S4D2 > S3D1 > S6D1 > S4D1. Under the S3 row spacing, peak LAI values for each planting density were 3.08, 3.48, and 3.82, respectively. Compared to D1 and D2, the LAI for the D3 density increased by 24.13% and 9.74%, respectively (*p*< 0.05). Under the S4 row spacing, peak LAI values for the three planting densities were 2.83, 3.11, and 4.45, respectively. In comparison to D1 and D2, the D3 density resulted in LAI increases of 56.88% and 42.55%, respectively (*p*< 0.05). Under the S6 row spacing, peak LAI values for the three planting densities were 2.94, 3.23, and 4.65, respectively. Relative to D1 and D2, the D3 density led to LAI increases of 58.15% and 43.68%, respectively (*p*< 0.05).

### 3.2 Dry matter accumulation

**Figure 4:**
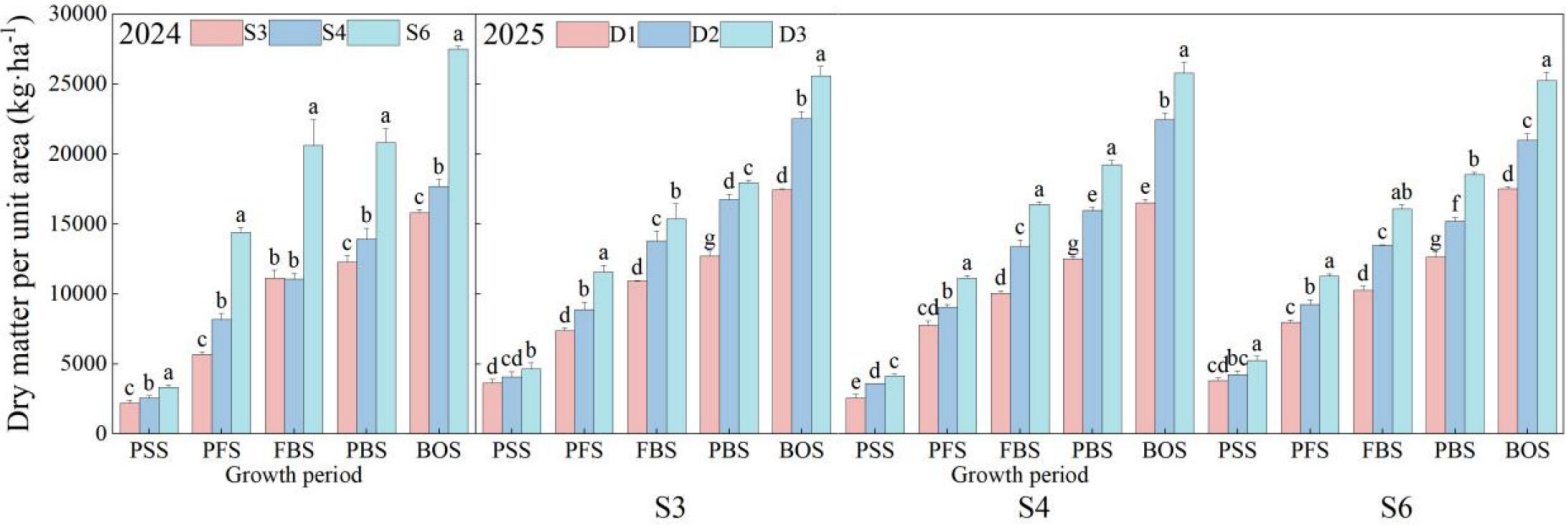
Dynamics of population-level dry matter accumulation in cotton under different row spacing configurations and planting densities. PSS, Peak squaring stage; PFS, Peak flowering stage; FBS, Flowering and boll stage; PBS, Peak boll-setting stage; BOS, Boll opening stage. Different letters indicate a significant difference at *p*< 0.05 among treatments.

As illustrated in **Figure 4**, the total dry matter mass of cotton exhibited a rapid increase throughout the growth stages over both years. Additionally, dry matter accumulation demonstrated a significant upward trend in response to higher planting densities (*p*< 0.05). During the peak squaring stage, however, dry matter accumulation occurred slowly across all treatments. In contrast, from the peak flowering stage to the boll opening stage, dry matter accumulation accelerated markedly, achieving its maximum value in all treatments by the boll opening stage. Under the same row spacing, differences in dry matter accumulation among the treatments were not significant prior to the squaring stage. However, as the growth stages advanced, these differences became increasingly pronounced. The D3 density consistently exhibited the highest level of dry matter accumulation throughout the entire growth period in both years, with annual patterns of dry matter variation remaining consistent. In 2024, among the three-row spacing treatments, the total population dry matter accumulation was greatest in the S6 treatment, with significant differences noted across all treatments (*p*< 0.05). By the boll opening stage, dry matter accumulation under S6 reached 27,492.93 kg·ha^-1^, surpassing that of S3 and S4 by 74.05% and 55.53%, respectively (*p*< 0.05). In 2025, across all three row spacings, the D3 planting density consistently yielded the highest total population dry matter accumulation. At the boll opening stage, dry matter accumulation under the S3D3 treatment reached 25,579.87 kg·ha^-1^, which was 46.56% and 13.48% higher than that of the S3D1 and S3D2 treatments, respectively (*p*< 0.05). The S4D3 treatment attained 25,789.00 kg·ha^-1^, exceeding S4D1 and S4D2 by 56.25% and 14.77%, respectively (*p*< 0.05). The S6D3 treatment achieved 25,264.25 kg·ha^-1^, surpassing S6D1 and S6D2 by 44.15% and 20.46%, respectively (*p*< 0.05).

### 3.3 Dry matter distribution

**Figure 5:**
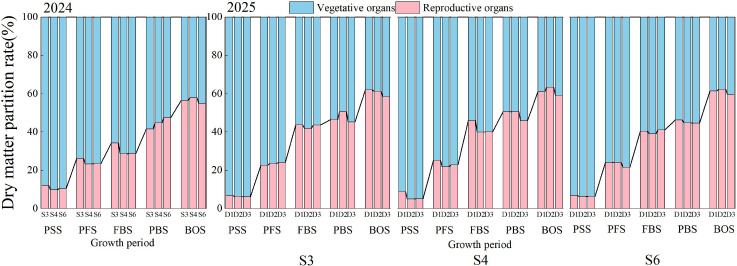
Proportional distribution of dry matter in cotton under different row spacing configurations and planting densities. PSS, Peak squaring stage; PFS, Peak flowering stage; FBS, Flowering and boll stage; PBS, Peak boll-setting stage; BOS, Boll opening stage.

As illustrated in **Figure 5**, the proportion of vegetative organs in cotton plants progressively declined with advancing growth stages across all treatments over the two-year period, while the proportion of reproductive organs exhibited an inverse trend. In 2024, the proportion of vegetative organs decreased from 87.92% - 90.16% to 42.19% - 45.13%, whereas the proportion of reproductive organs increased from 9.84% - 12.08% to 54.87% - 57.81%. Similarly, in 2025, the proportion of vegetative organs declined from 91.10% - 94.96% to 36.68% - 41.67%, while the proportion of reproductive organs rose from 5.04% - 8.90% to 58.33% - 63.32%. At the same growth stage, planting density significantly influences the allocation ratio between vegetative and reproductive organs (*p*< 0.05). Across various growth stages, the distribution pattern varies distinctly among treatments. Data from 2024 indicate that no significant differences were observed among the treatments at the peak squaring stage. At the peak boll-setting and boll opening stages, significant differences (*p*< 0.05) were observed among the treatments. From the peak squaring to the peak boll-setting stage, the S4 treatment demonstrated the highest proportion of dry matter allocation to vegetative organs, while the S3 treatment exhibited the greatest allocation to reproductive organs. During the boll opening stage, the S6 treatment allocated the largest proportion to vegetative organs, whereas the S4 treatment allocated the most to reproductive organs. A comparison of the 2025 data indicated that the allocation ratios to vegetative organs among the three row spacing treatments did not show significant differences prior to the flowering and boll stages. However, significant differences emerged among the treatments from the peak boll-setting stage to the boll opening stage (*p*< 0.05). Around the flowering and boll stages, cotton enters a phase characterized by concurrent vegetative and reproductive growth, resulting in a more balanced allocation between these organs. Following the conclusion of flowering, cotton shifts into a period of rapid reproductive growth, during which reproductive development predominates. Consequently, the proportion allocated to reproductive organs continues to rise, while the allocation to vegetative growth gradually decreases. Therefore, an appropriate planting density enhances the accumulation of reproductive organs and promotes a balanced allocation ratio, thereby improving the coordination between vegetative and reproductive development and contributing to the overall growth and productivity of cotton.

### 3.4 Leaf nitrogen content

**Figure 6:**
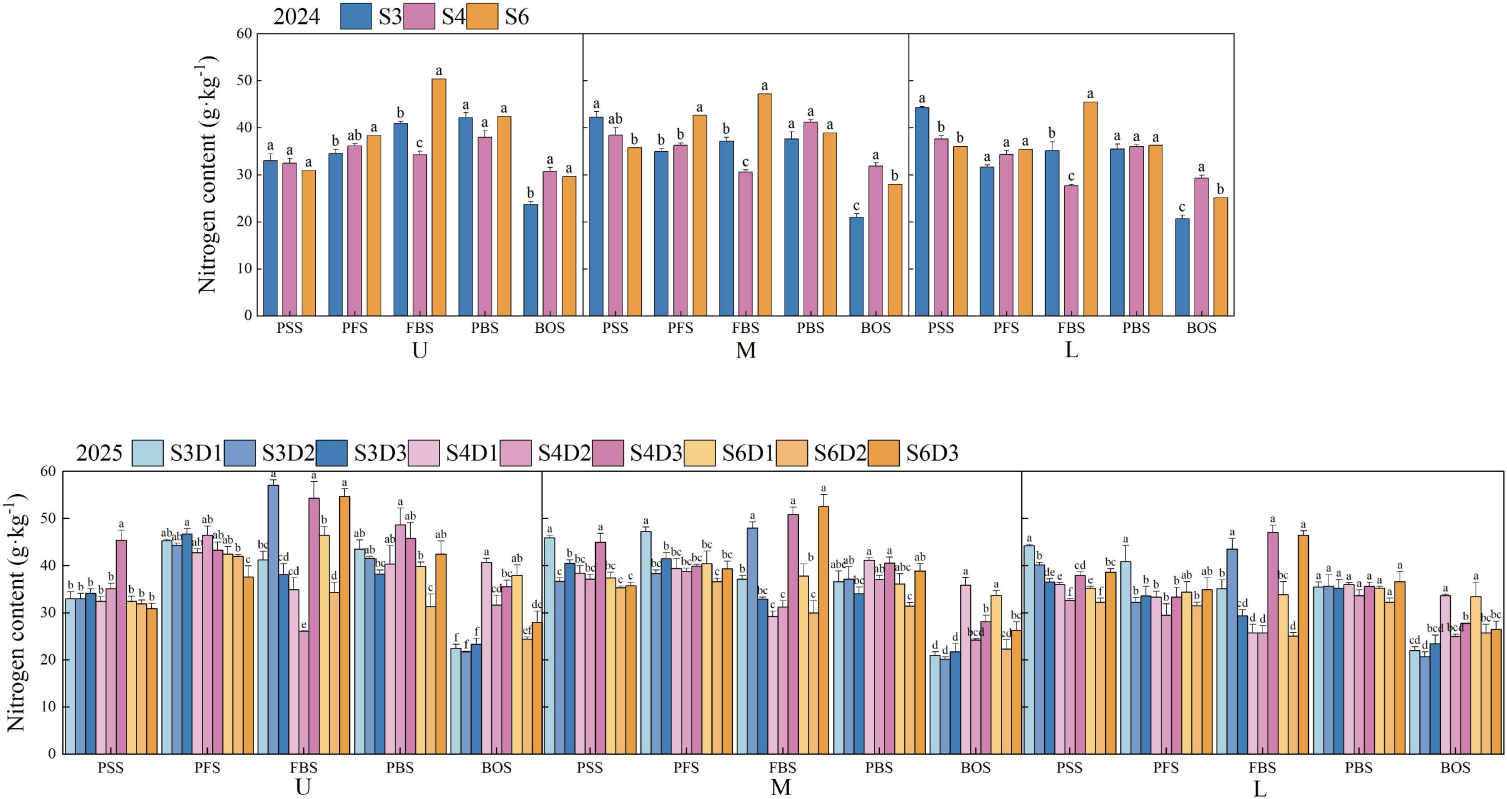
Changes in leaf nitrogen content of cotton under different row spacings and planting densities. PSS, Peak squaring stage; PFS, Peak flowering stage; FBS, Flowering and boll stage; PBS, Peak boll-setting stage; BOS, Boll opening stage. U, M, and L indicate the upper part of the canopy, the middle part of the canopy, and the lower part of the canopy, respectively. Different letters indicate a significant difference at *p*< 0.05 among treatments.

**Figure 6** illustrates the variations in nitrogen content of cotton leaves across different treatments, revealing consistent overall trends over two years. The nitrogen content in upper leaves initially increased before declining with growth, whereas the nitrogen content in middle and lower leaves decreased gradually. Row spacing and planting density significantly influenced these changes (*p*< 0.05). In 2024, during the flowering and boll stage, the nitrogen content in upper, middle, and lower leaves was highest under the S6 treatment, significantly exceeding that of the S3 and S4 treatments (*p*< 0.05), with increases of 23.29%, 47.09%, 27.14%, 54.41%, 29.52%, and 64.03%, respectively. At the boll opening stage, the S4 treatment exhibited the highest nitrogen content, surpassing S3 and S6 treatments by 29.28%, 3.56%, 51.64%, 13.92%, 41.80%, and 16.61%, respectively. In 2025, during the flowering and boll stage, under the S3 treatment, the D2 density demonstrated significantly higher nitrogen content in upper, middle, and lower leaves compared to D1 and D3 (*p*< 0.05), with increases of 40.93%, 52.33%, 29.26%, 45.81%, 23.93%, and 48.10%. Under the S4 treatment, the D3 density was significantly greater than D1 and D2 (*p*< 0.05), with increases of 58.33%, 112.33%, 74.00%, 63.03%, 83.03%, and 82.80%. Under the S6 treatment, the D3 density also significantly exceeded D1 and D2 (*p*< 0.05), with increases of 17.79%, 59.45%, 39.08%, 75.30%, 37.13%, and 85.55%. During the boll opening stage, the nitrogen content in the upper leaves was ranked as follows: S4D1 > S6D1 > S4D3 > S4D2 > S6D3 > S6D2 > S3D3 > S3D1 > S3D2. For the middle leaves, the order was: S4D1 > S6D1 > S4D3 > S6D3 > S4D2 > S6D2 > S3D3 > S3D1 > S3D2. In the lower leaves, the ranking was: S4D1 > S6D1 > S4D3 > S6D3 > S6D2 > S4D2 > S3D3 > S3D1 > S3D2.

### 3.5 Leaf phosphorus content

**Figure 7:**
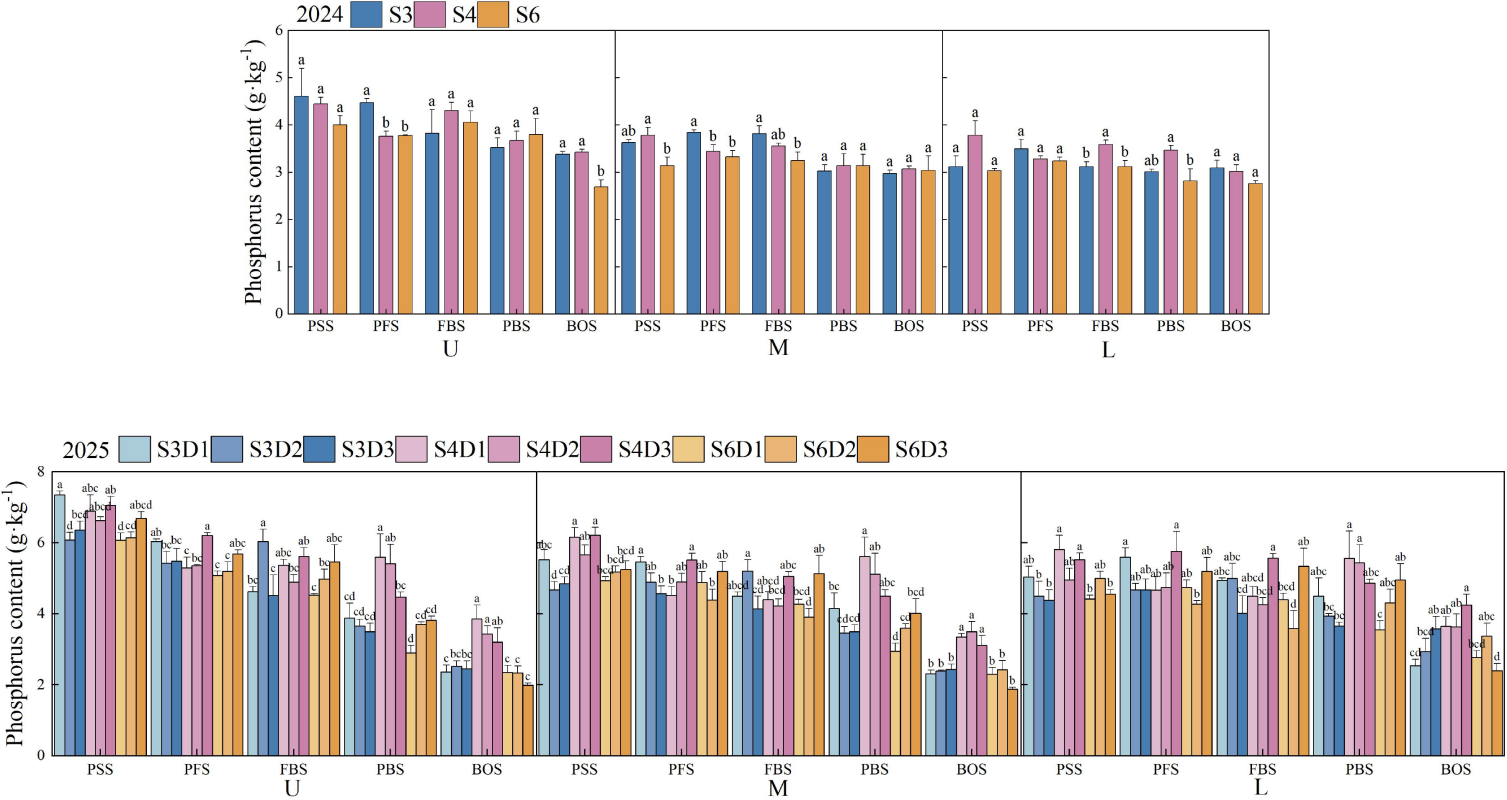
Changes in leaf phosphorus content of cotton under different row spacings and planting densities. PSS, Peak squaring stage; PFS, Peak flowering stage; FBS, Flowering and boll stage; PBS, Peak boll-setting stage; BOS, Boll opening stage. U, M, and L indicate the upper part of the canopy, the middle part of the canopy, and the lower part of the canopy, respectively. Different letters indicate a significant difference at *p*< 0.05 among treatments.

**Figure 7** illustrates the changes in phosphorus content in cotton leaves across various treatments, revealing consistent overall trends over two years. The phosphorus content in the upper, middle, and lower leaves of all treatments gradually decreased with growth, and both row spacing and planting density significantly influenced this outcome (*p*< 0.05). In 2024, the S3 treatment exhibited the highest phosphorus content in the upper and middle leaves during the peak flowering stage, significantly surpassing the levels observed in S4 and S6 (*p*< 0.05), with increases of 18.85%, 18.45%, 11.69%, and 15.44%, respectively. At the flowering and boll stage, the S4 treatment demonstrated the highest phosphorus content in the lower leaves, significantly exceeding that of S3 and S6 (*p*< 0.05), with increases of 15.04% and 15.15%, respectively. In 2025, under the S3 treatment, D1 density exhibited the highest overall leaf phosphorus content, while D2 density recorded the highest levels during the flowering and boll stage, with increases of 30.68%, 33.70%, 15.71%, 25.75%, 0.97%, and 24.52% compared to D1 and D3, respectively. Under the S4 treatment, D3 density was highest from the peak squaring stage to the flowering and boll stage, with increases of 4.69%, 14.83%, 12.88%, 19.62%, 23.73%, and 30.86% over D1 and D2 at the flowering and boll stage. In contrast, D1 density was highest after peak boll-setting stage, with increases of 3.50%, 25.11%, 9.70%, 24.95%, 2.36%, and 14.32% compared to D2 and D3 at the peak boll-setting stage. Under the S6 treatment, D3 density consistently exhibited the highest leaf phosphorus content overall; during the flowering and boll stage, it was 20.85%, 9.67%, 20.22%, 31.57%, 21.29%, and 48.93% higher than D1 and D2, respectively. At the boll opening stage, the order of phosphorus content was as follows: for upper leaves, S4D1 > S4D2 > S4D3 > S3D2 > S3D3 > S3D1 > S6D1 > S6D2 > S6D3; for middle leaves, S4D2 > S4D1 > S4D3 > S3D3 > S6D2 > S3D2 > S3D1 > S6D1 > S6D3; and for lower leaves, S4D3 > S4D1 > S4D2 > S3D3 > S6D2 > S3D2 > S6D1 > S3D1 > S6D3.

### 3.6 Leaf potassium content

**Figure 8:**
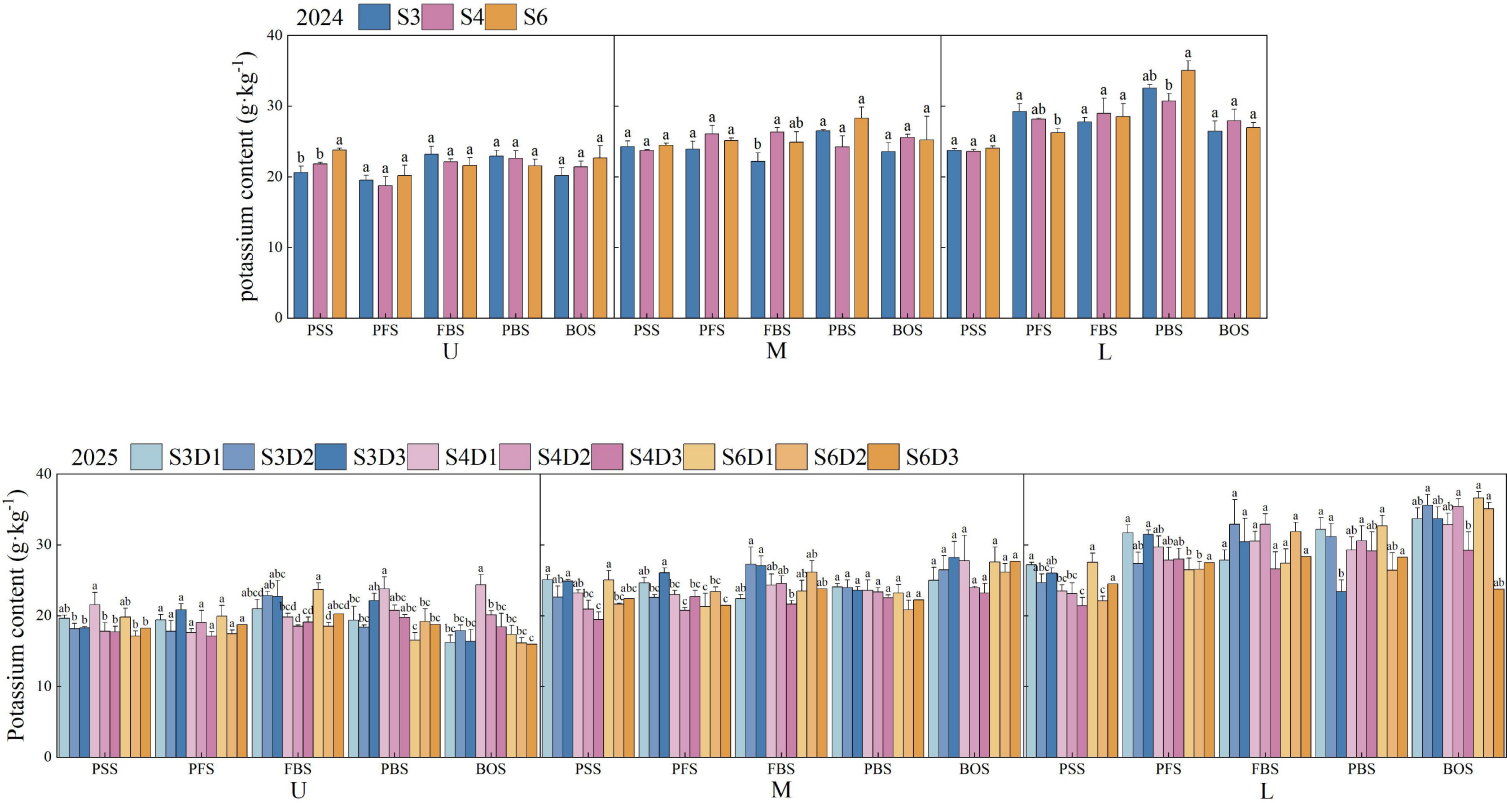
Changes in leaf potassium content of cotton under different row spacings and planting densities. PSS, Peak squaring stage; PFS, Peak flowering stage; FBS, Flowering and boll stage; PBS, Peak boll-setting stage; BOS, Boll opening stage. U, M, and L indicate the upper part of the canopy, the middle part of the canopy, and the lower part of the canopy, respectively. Different letters indicate a significant difference at *p*< 0.05 among treatments.

As illustrated in **Figure 8**, row spacing and planting density significantly influenced the potassium content in cotton leaves (*p*< 0.05). In 2024, the potassium content in the upper, middle, and lower leaves initially increased and subsequently decreased throughout the growth process. In 2025, the potassium content in the upper leaves exhibited a similar pattern, increasing initially before declining, while the middle and lower leaves showed a gradual increase. During the peak squaring stage in 2024, the potassium content in the upper leaves under the S6 treatment was significantly greater than that under the S3 and S4 treatments (*p*< 0.05), with increases of 15.37% and 9.04%, respectively. At the peak boll-setting stage, the potassium content in the lower leaves under the S6 treatment was significantly higher than that under the S4 treatment (*p*< 0.05), exceeding the levels in the S3 and S4 treatments by 7.66% and 14.01%, respectively. In 2025, across different growth stages, the potassium content in the lower leaves of all treatments surpassed that in the middle and upper leaves; within the same row spacing treatment, no significant differences were observed among the various densities. At the boll opening stage, the potassium content in upper leaves across all treatments was ranked as follows: S4D1 > S4D2 > S4D3 > S3D2 > S6D1 > S3D3 > S3D1 > S6D2 > S6D3. In middle leaves, the order was: S3D3 > S4D1 > S6D3 > S6D1 > S3D2 > S6D2 > S3D1 > S4D2 > S4D3. For lower leaves, the ranking was: S6D1 > S3D2 > S4D2 > S6D2 > S3D3 > S3D1 > S4D1 > S4D3 > S6D3.

### 3.7 Yield and nutrients

The data presented in the **Table 1** indicate that planting density and row spacing configurations significantly influence the accumulation of nitrogen (N), phosphorus (P), and potassium (K) in leaves at various canopy positions (upper, middle, and lower) (*p*< 0.05). The nutrient content of leaves across all treatments in both years exhibited consistent patterns during the boll opening stage. Specifically, the N content in upper leaves was greater than that in middle and lower leaves, whereas the P and K contents in upper and middle leaves were lower than those in lower leaves. In 2024, treatment S3 demonstrated the lowest N content across upper, middle, and lower leaves, which was significantly less than that observed in treatments S4 and S6 (*p*< 0.05). The reductions were 29.28%, 24.84%, and 51.64% for upper, middle, and lower leaves, respectively, compared to S4, and 24.88%, 41.80%, and 21.60% compared to S6. In 2025, the D2 density under the S3 row spacing produced the highest yield, whereas the D3 density exhibited the greatest concentrations of nitrogen (N), phosphorus (P), and potassium (K) in the upper, middle, and lower leaves. Similarly, under the S4 row spacing, the D2 density also achieved the highest yield; however, the D1 density demonstrated the highest nutrient content in leaves across all canopy positions. Under the S6 row spacing, the D2 density again yielded the highest output, while the D1 density, consistent with other row spacing treatments, showed elevated nutrient contents. Although a consistent vertical gradient in leaf nutrient content was observed across various treatments, the optimal combination of row spacing and density for maximizing yield did not consistently coincide with the maximization of nutrient content in each canopy layer.

**Table 1:** Effects of row spacing and planting density on leaf nutrient content at boll opening stage.

Year	Row spacing	Planting density	Seed cotton yield (kg·ha^-1^)	U			M			L		
				N (g·kg^-1^)	P (g·kg^-1^)	K (g·kg^-1^)	N (g·kg^-1^)	P (g·kg^-1^)	K (g·kg^-1^)	N (g·kg^-1^)	P (g·kg^-1^)	K (g·kg^-1^)
2024	S3		5322.43 c	23.75 b	3.38 a	20.13 a	21.01 c	2.97 a	23.56 a	20.66 c	3.09 a	26.45 a
	S4		5490.32 b	30.71 a	3.43 a	21.40 a	31.86 a	3.07 a	25.58 a	29.29 a	3.02 a	27.94 a
	S6		5714.20 a	29.65 a	2.69 b	22.68 a	27.97 b	3.04 a	25.23 a	25.12 b	2.76 a	26.95 a
2025	S3	D1	5395.45 bc	22.48 f	2.36 c	16.21 bc	21.01 d	2.30 b	24.99 a	21.99 cd	2.54 cd	33.71 ab
		D2	5960.20 ab	21.73 f	2.52 bc	17.88 bc	20.18 d	2.38 b	26.47 a	20.69 d	2.93 bcd	35.60 a
		D3	5220.33 c	23.32 f	2.45 bc	16.34 bc	21.75 d	2.43 b	28.19 a	23.47 bcd	3.58 ab	33.72 ab
	S4	D1	5493.40 bc	40.71 a	3.86 a	24.38 a	35.86 a	3.34 a	27.74 a	33.62 a	3.64 ab	32.88 ab
		D2	6080.12 ab	31.64 cd	3.43 a	20.12 b	24.19 bcd	3.49 a	23.96 a	24.99 bcd	3.63 ab	35.40 a
		D3	5900.65 abc	35.54 bc	3.20 ab	18.42 bc	28.12 b	3.11 a	23.20 a	27.75 b	4.25 a	29.25 b
	S6	D1	5580.52 bc	37.98 ab	2.34 c	17.36 bc	33.76 a	2.30 b	27.56 a	33.48 a	2.77 bcd	36.60 a
		D2	6420.47 a	24.42 ef	2.33 c	16.11 bc	22.34 cd	2.42 b	26.14 a	25.77 bc	3.37 abc	35.07 a
		D3	5605.11 bc	27.99 de	1.99 c	15.95 c	26.30 bc	1.87 b	27.65 a	26.45 bc	2.39 d	23.75 c
Source of variation												
Row Spacing			ns	**	**	**	**	**	ns	**	**	ns
Planting Density			**	**	ns	ns	**	ns	ns	**	ns	**
Row Spacing × Planting Density			ns	**	ns	ns	**	ns	ns	ns	ns	*

Different lowercase letters within the same column indicate significant differences (*p*< 0.05). “ns” indicates no significant effect; * indicates a significant effect (*p*< 0.05); ** indicates a highly significant effect (*p*< 0.01). S and D refer to different row spacing configurations and different planting densities, respectively. U, M, and L indicate the upper part of the canopy, the middle part of the canopy, and the lower part of the canopy, respectively.

### 3.8 Spatial distribution and boll quality of cotton

**Figure 9:**
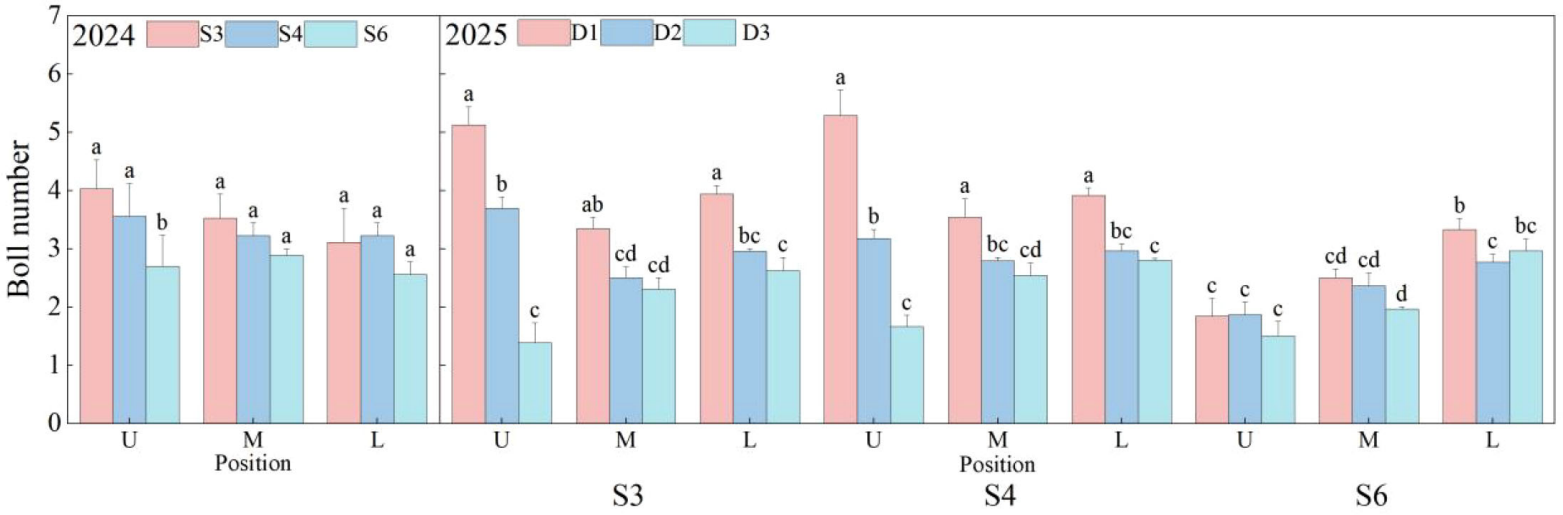
Effects of row spacing configurations and planting densities on the spatial distribution of cotton bolls. U, M, and L indicate the upper part of the canopy, the middle part of the canopy, and the lower part of the canopy, respectively. Different letters indicate a significant difference at *p*< 0.05 among treatments.

As depicted in **Figure 9**, the spatial distribution of cotton bolls was significantly influenced by row spacing and planting density (*p*< 0.05), with notable variations in boll counts between the middle and upper canopy. In 2024, the S3 treatment exhibited the highest upper-canopy boll count at 4.02, exceeding S4 and S6 by 13.28% and 49.49%, respectively. In 2025, upper-canopy bolls in the S3 and S4 treatments were generally more abundant than those in S6 across all densities; specifically, the D1 density significantly enhanced boll numbers in all canopy layers under S3 and S4 (*p*< 0.05), whereas no density-related differences were observed under S6. The S3D1 and S4D1 treatments yielded the highest boll counts across all positions, suggesting that lower densities promoted upper-canopy boll formation under S3 and S4. No significant differences in boll numbers in the middle and lower canopy were found between D2 and D3 across all row spacings. Overall, optimized row spacing and planting density may enhance boll development, create an ideal canopy structure, and consequently improve cotton yield and quality.

**Figure 10:**
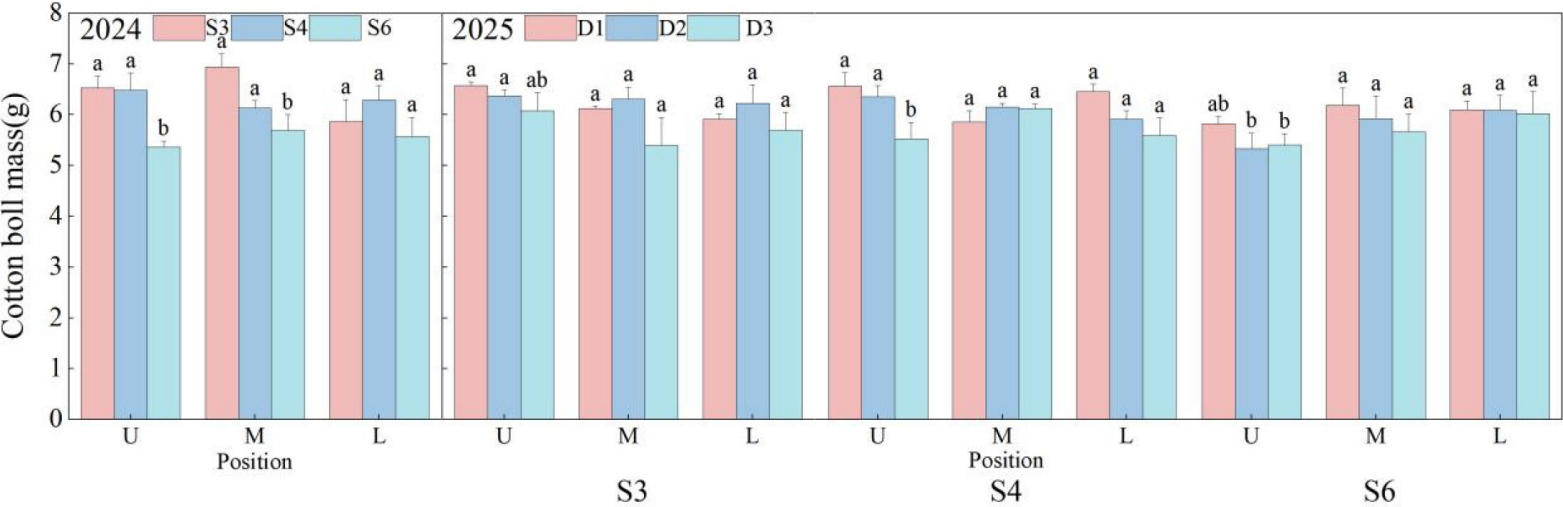
Effects of row spacing configurations and planting densities on cotton boll mass. U, M, and L indicate the upper part of the canopy, the middle part of the canopy, and the lower part of the canopy, respectively. Different letters indicate a significant difference at *p*< 0.05 among treatments.

As shown in **Figure 10**, row spacing and planting density did not significantly influence cotton boll quality in the middle and lower canopy layers; however, planting density had a marginal effect on boll quality in the upper canopy. In 2024, the S3 and S4 treatments demonstrated superior performance compared to S6 in terms of upper and middle canopy boll quality, with respective increases of 21.74% and 20.96% for the upper canopy, and 22.00% and 7.76% for the middle canopy. In 2025, D2 enhanced middle and lower canopy quality under S3, while D3 negatively impacted upper canopy quality. Under S4, D1 exhibited the highest upper canopy quality, surpassing D2 and D3 by 3.36% and 18.75%, respectively. Under S6, D1 achieved the best boll quality across all layers, with upper canopy quality 9.13% and 7.71% higher than that of D2 and D3.

### 3.9 Cotton yield

As shown in **Table 2**, the two-year data on bolls per plant indicated that both row spacing and planting density significantly influenced this trait (*p*< 0.05). In 2024, the S3 and S4 treatments yielded significantly more bolls per plant than the S6 treatment, with increases of 62.44% compared to the latter (*p*< 0.05). In 2025, a significant interaction effect was observed between row spacing and planting density, resulting in notable differences among all treatments (*p*< 0.05). Throughout both years, a consistent trend emerged, demonstrating that the number of bolls per plant gradually decreased as planting density increased. Additionally, significant variations in single boll weight were identified among the treatments (*p*< 0.05). In 2024, the S3 and S4 treatments exhibited significantly higher single boll weights compared to the S6 treatment, with increases of 5.18% and 4.64%, respectively (*p*< 0.05). In 2025, the S6D2 treatment achieved the highest single boll weight of 5.97 g, which was significantly 12.43% greater than that of the S6D3 treatment (*p*< 0.05); no significant differences were observed among the remaining treatments. Lint percentage showed no significant variations across all treatments over the two experimental years. However, in 2025, the D2 density treatment consistently yielded higher lint percentages than the D1 and D3 density treatments across all three row spacing regimes. As indicated in the table, planting density had a highly significant effect on seed cotton yield (*p*< 0.05). In 2024, the S6 treatment produced significantly higher seed cotton yields than the S3 and S4 treatments, exceeding them by 7.36% and 4.08%, respectively (*p*< 0.05). In 2025, the seed cotton yield of the treatments followed this descending order: S6D2 > S4D2 > S3D2 > S4D3 > S6D3 > S6D1 > S4D1 > S3D1 > S3D3. Across all three row spacing regimes, the D2 density treatment consistently achieved the highest yields, with S3D2, S4D2, and S6D2 reaching 5960.20 kg·ha^-^¹, 6080.12 kg·ha^-^¹, and 6420.47 kg·ha^-^¹, respectively. These findings indicate that a moderate planting density is optimal for all three row spacing configurations, while excessively low or high densities tend to limit yield potential.

**Table 2:** Effects of row spacing configurations and planting densities on cotton yield and its components.

Year	Row spacing	Planting density	Boll number per plant	Cotton boll mass (g)	Seed cotton yield (kg·ha^-1^)	Lint percentage (%)
2024	S3		10.77 a	5.89 a	5322.43 c	42.23 a
	S4		10.77 a	5.86 a	5490.32 b	42.51 a
	S6		6.63 b	5.60 b	5714.20 a	42.77 a
2025	S3	D1	12.42 a	5.80 ab	5395.45 bc	40.67 a
		D2	9.16 b	5.74 ab	5960.20 ab	42.00 a
		D3	6.32 d	5.77 ab	5220.33 c	40.67 a
	S4	D1	12.40 a	5.78 ab	5493.40 bc	41.33 a
		D2	8.94 b	5.83 ab	6080.12 ab	41.67 a
		D3	7.01 d	5.70 ab	5900.65 abc	41.33 a
	S6	D1	8.00 c	5.67 ab	5580.52 bc	41.33 a
		D2	6.67 d	5.97 a	6420.47 a	42.00 a
		D3	6.43 d	5.31 b	5605.11 bc	41.00 a
Source of variation						
Row Spacing			**	ns	ns	ns
Planting Density			**	ns	**	ns
Row Spacing × Planting Density			**	ns	ns	ns

Different lowercase letters within the same column indicate significant differences (*p*< 0.05). “ns” indicates no significant effect; * indicates a significant effect (*p*< 0.05); ** indicates a highly significant effect (*p*< 0.01). S and D refer to different row spacing configurations and different planting densities, respectively.

## 4 Discussion

LAI is a critical quantitative parameter for assessing crop population development. It not only characterizes the photosynthetic potential of the canopy but also serves as a vital indicator of overall crop growth vigor. Previous studies have demonstrated that planting density significantly influences LAI, exhibiting a positive correlation; specifically, LAI increases with higher planting densities (Darawsheh et al., 2009). Moreover, variations in density gradients can substantially affect both the magnitude of the LAI peak and the timing of its occurrence (Zuo et al., 2023). The findings of this study align with these conclusions. Across the three row spacing configurations, LAI increased markedly with rising planting density, achieving its maximum value at the D3 density. Additionally, earlier research has indicated that, despite differences in row spacing configurations, the overall trend of cotton LAI remains consistent, typically peaking at the full boll stage and subsequently declining as the boll opening stage begins (Brodrick et al., 2012; Zhang et al., 2021). The experimental results of this study across the three row spacing configurations further corroborate and enhance these previous findings, revealing that cotton populations under high-density planting sustained a relatively high LAI level throughout the entire growth period. Despite variations in row spacing configurations, the overall trend of cotton leaf area index (LAI) aligned with findings from previous studies, peaking at the full boll stage and subsequently declining during the boll opening stage. Previous research has indicated that the increase in LAI, coupled with reduced light transmittance, is a fundamental challenge associated with high-density planting (Hu et al., 2021; Zuo et al., 2024). In contrast, this study found that the high-density cotton population sustained a relatively high LAI during the late growth stage without significant degradation. This phenomenon is likely due to the optimized microclimate created by the row spacing configuration. These results offer theoretical support for the coordinated management of planting density and row spacing in the development of high-yield cotton canopies.

Previous studies have shown that dry matter accumulation, as the final product of crop photosynthesis, and its translocation efficiency are critical for crop yield formation (Khan et al., 2020). Additionally, crop dry matter accumulation varies significantly due to multiple factors, including the genetic characteristics of varieties and cultivation environments (Marshall and Ohm, 1987; Teich et al., 1993). Moreover, an appropriate planting pattern can enhance dry matter accumulation in cotton, optimize the distribution ratio of dry matter among different organs, and increase the proportion of dry matter in reproductive organs, thereby supporting high yield (Ding et al., 2024). The findings of this study align with these previous results and further elucidate the regulatory effects of planting density and row spacing on dry matter accumulation in cotton populations. Under the three row spacing treatments, total dry matter accumulation increased with higher planting density, with the maximum observed in the D3 density treatment. Among the various row spacing configurations, the S6 wide-narrow row planting pattern resulted in significantly greater dry matter accumulation in cotton compared to other treatments. This pattern suggests that a rational combination of planting density and row spacing can enhance dry matter accumulation by optimizing population structure. Previous studies have established that total dry matter accumulation in populations increases with higher planting densities (Chen et al., 2022; Ethridge et al., 2022). This conclusion has been corroborated through cultivation experiments across various crops, underscoring the universal regulatory influence of planting density on dry matter accumulation. The findings of this study demonstrate that population dry matter accumulation rises with planting density across all three row spacing treatments, with the highest value observed in the D3 density treatment. This phenomenon may arise from the increase in planting density, which broadens the population base. This expansion enhances the canopy’s capacity for light interception and improves light use efficiency, ultimately leading to a greater accumulation of photosynthetic products at the population level. These results align with previous conclusions, further affirming the substantial impact of planting density on dry matter accumulation in cotton populations.

Previous studies have elucidated the characteristics of nitrogen absorption, distribution, and dynamic changes across different cotton organs. Throughout the entire growth period, variations in nitrogen absorption are observed among various cotton organs, with leaves exhibiting the highest nitrogen absorption ratio, followed by roots and stems. As the growth process advances to the boll opening stage, the total nitrogen content in roots, stems, and leaves demonstrates a rapid decline (Chen et al., 2024; Clawson et al., 2006; Nie et al., 2019). The findings of this study align with the previously discussed dynamic variation characteristics of leaf nitrogen content and further elucidate the spatial and temporal distribution patterns of nutrient content in cotton leaves. The nitrogen content in upper cotton leaves initially increased before declining as growth stages progressed, whereas the nitrogen content in middle and lower leaves exhibited a gradual decrease. The phosphorus content in leaves across all positions diminished steadily throughout the growth period. In contrast, the potassium content in leaves varied significantly over the two years; however, the lower leaves consistently exhibited higher potassium levels, which continued to rise as growth stages advanced. According to the research by (Iqbal et al., 2020) and (Chen et al., 2019), which indicates that planting density enhances leaf nutrient accumulation, this spatial variation in nutrient distribution may serve as a self-regulatory mechanism for cotton plants. This mechanism likely facilitates the translocation of nutrients to the upper functional leaves, thereby ensuring optimal photosynthetic efficiency in the late growth stage.

Previous studies have shown that seed cotton yield is jointly determined by boll weight and the total number of bolls per unit area, with significant influences from environmental factors and cultivation management practices. Planting density, as a core regulatory measure, plays a crucial role in yield formation. Specifically, boll weight exhibits a negative correlation with planting density, whereas the number of bolls per unit area initially increases and subsequently decreases as planting density rises. Additionally, planting density serves as the primary factor influencing the number of bolls per plant, resulting in a downward-opening quadratic curve relationship between seed cotton yield and planting density (Kimura et al., 2024; Ma et al., 2025). The findings of this study align with previous conclusions and further elucidate the influence of planting density on cotton yield and its components. Specifically, the number of bolls per plant declines as planting density increases. Additionally, seed cotton yield initially rises before subsequently decreasing with higher planting densities. Notably, planting density significantly affects the final seed cotton yield. This pattern indicates that achieving a high cotton yield necessitates an appropriate range of planting density; excessively high density hinders boll formation per plant, while excessively low density does not ensure an adequate number of bolls per unit area. Only an optimal planting density can effectively balance these two factors to maximize yield. Previous studies on the synergistic effects of planting patterns and density have demonstrated that various row spacing configurations exert distinct regulatory influences on cotton yield and quality (Zuo et al., 2024). Specifically, wide-row high-density planting can achieve high yields by increasing boll weight and exhibits superior defoliation performance. This characteristic effectively reduces leaf impurities in machine-harvested cotton, thereby enhancing the quality of machine harvesting (Wang et al., 2019), In contrast, the 1-film 3-row planting reduces harvest density compared to the 1-film 6-row planting while optimizing canopy structure. This adjustment significantly improves light transmittance in the upper and middle sections of the canopy from the germination stage to the flowering stage, resulting in an increased number of bolls and boll weight in these areas, which compensates for yield losses associated with lower density (Gao et al., 2024). Excessively high planting density results in decreased boll weight, increased boll abscission, delayed maturity, and ultimately leads to reduced yield (Khan et al., 2019), Conversely, low density may enhance boll weight but does not significantly improve yield due to an inadequate number of bolls per unit area. This study further elucidates the coordinated regulatory effects of row spacing and planting density. At the same planting density, the number of bolls per plant in the 1-film 3-row and 1-film 4-row treatments was significantly greater than that in the 1-film 6-row treatment. However, no significant differences in boll weight were observed among the various treatments at the same density. Integrating the findings of (Gao et al., 2024), which indicate that the 1-film 3-row planting enhances yield by optimizing canopy structure, it can be concluded that appropriate row spacing configurations (1-film 3-row and 1-film 4-row) mitigate the adverse effects of planting density by increasing the number of bolls per plant. This conclusion aligns with (Khan et al., 2019), who asserted that “excessively high density leads to yield reduction,” and supports the perspective of (Wang et al., 2019) that “synergistic optimization of planting pattern and density can improve yield.” These findings provide theoretical support for the precise configuration of high-yield cotton planting patterns.

## 5 Conclusion

Under the three row spacing configurations tested in this experiment, both the leaf area index and population dry matter accumulation increased significantly with higher planting density. As growth stages progressed, the proportion of dry matter allocated to reproductive organs gradually increased. Leaf nitrogen (N) content in cotton was significantly affected by row spacing and planting density. Upper-leaf N increased initially then decreased with growth stages, while N in middle and lower leaves declined slowly. Leaf phosphorus (P) content at all three positions decreased gradually with plant development. Upper-leaf potassium (K) content showed a similar trend to N, rising first then falling. The number of bolls per plant decreased as planting density increased, while seed cotton yield was significantly influenced by density. Across all three row spacings, the highest yield was recorded at a medium density of 180,000 plants ha^-1^, with the S6D2 treatment yielding a maximum of 6420.47 kg·ha^-1^. In summary, under the 6-row-per-film planting pattern, a density of 180,000 plants ha^-1^ can enhance cotton yield.

## Data Availability

The original contributions presented in the study are included in the article/supplementary material. Further inquiries can be directed to the corresponding authors.
